# Delayed Heterogeneous Left Buttock Mass Following Gluteal Augmentation in a Young Symptomatic African American Woman: A Case Report

**DOI:** 10.7759/cureus.113740

**Published:** 2026-07-31

**Authors:** Bom K Bong, Trevor Williams, Nazia Khaleel, Makeda Green, Frederick Tiesenga

**Affiliations:** 1 Medical School, Caribbean Medical University School of Medicine, Willemstad, CUW; 2 Surgery, St. George’s University School of Medicine, St. George's, GRD; 3 Surgery, St. George's University School of Medicine, St. George's, GRD; 4 General Surgery, West Suburban Medical Center, Chicago, USA

**Keywords:** brazilian butt lift, chronic inflammation, cosmetic surgery complication, fat necrosis, gluteal augmentation, postoperative complication, sterile abscess, subcutaneous mass

## Abstract

A 41-year-old African-American woman with a history of gluteal fat grafting procedure, also known as a “Brazilian butt-lift (BBL),” approximately seven years prior, with subsequent revision two years later, presented five years after the revision with several months of painful swelling of the left buttock with palpable gluteal mass. Surgery was done to remove a 5.0 x 3.5 x 3.0 cm heterogeneous, yellow-tan soft tissue mass with yellow-green exudate. The mass was sent for biopsying revealing fibroadipose tissue with focal abscess formation, fat necrosis, calcification, and reactive changes on histology. Gram stain, bacterial cultures, and malignancy work-up were negative. These delayed sterile inflammatory changes are only some of the reported complications of gluteal surgical procedures. Our case is unique in that the development of the symptoms was delayed beyond the typical period expected after these types of procedures, and it highlights the importance of longitudinal post-procedural monitoring of patients undergoing these gluteal augmenting procedures to ensure adequate patient recovery and minimize adverse outcomes that may lead to further invasive procedures.

## Introduction

Cosmetic gluteal augmentation, more commonly referred to as a “Brazilian butt lift” (BBL), has become an increasingly popular procedure over recent years. It has become one of the most commonly performed cosmetic surgeries worldwide due to its ability to enhance gluteal contour using autologous fat grafting [1]. Despite how common the procedure has become, BBL procedures are associated with a variety of postoperative complications. These may range from minor contour irregularities to severe life-threatening events - the most common of which include seroma formation, infection, fat necrosis, calcification, contour deformities, chronic inflammatory reactions, and pulmonary fat embolism - the most serious complication associated with significant mortality rates [2]. As cosmetic gluteal augmentation procedures continue to rise in demand, recognition of delayed postoperative complications has become increasingly important.

Delayed inflammatory complications following gluteal fat grafting may present as painful soft tissue masses, sterile abscesses, or chronic nodular lesions that can clinically mimic infectious or malignant processes. These reactions may occur months to years after the original procedure and appear to be more frequently associated with revision surgeries or repeated fat grafting procedures [3]. Because imaging and physical examination findings are often nonspecific, definitive diagnosis often requires surgical excision with histopathologic evaluation to exclude malignancy and infectious etiologies [3].

This report describes a case of a delayed inflammatory gluteal mass occurring after a prior cosmetic gluteal augmentation. Pathologic evaluation ultimately demonstrated benign chronic inflammatory changes consistent with sterile postoperative soft tissue reactions. This case highlights the importance of recognizing chronic sterile inflammatory complications following cosmetic gluteal augmentation procedures and emphasizes the role of pathologic evaluation in distinguishing benign postoperative inflammatory lesions from infectious or neoplastic conditions.

## Case presentation

We present a female patient with a past medical history significant for childhood asthma, allergic rhinitis, obesity, and seasonal allergies who presented with several months of progressive pressure and compression sensation involving the left buttock and hip region. The patient denied tobacco use but endorsed moderate alcohol consumption and marijuana use. Her surgical history was notable for prior cosmetic gluteal augmentation, including a Brazilian butt lift (BBL) performed seven years earlier with revision surgery two years thereafter the initial surgery. She reported persistent discomfort localized to the left gluteal region that progressively worsened over time, prompting further evaluation. Imaging and clinical examination identified a left buttock subcutaneous mass with concern for a chronic inflammatory or soft tissue process, and operative excision was recommended.

Within the past year, the patient underwent excision of a left buttock subcutaneous mass with associated abscess measuring approximately 6 × 7 cm on clinical examination. The procedure was performed under general anesthesia. Estimated blood loss was minimal, and no intraoperative complications occurred.

**Figure 1:**
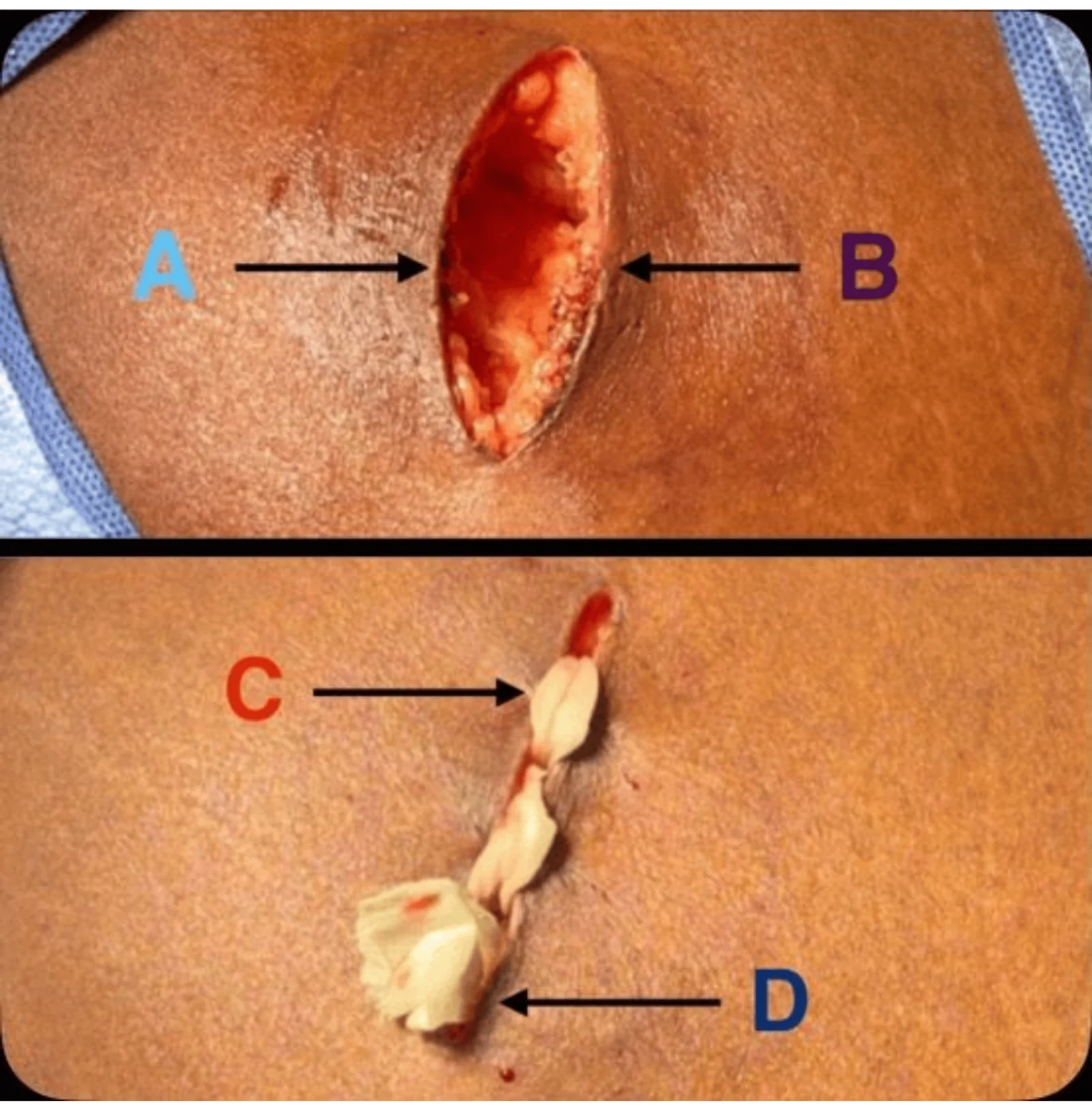
Arrows A and B demonstrate the linear incision over the left gluteal region following excision of the subcutaneous mass, whereas arrows C and D highlight the iodoform packing placed between the interrupted nylon sutures for postoperative wound management.

A linear incision was made over the palpable lesion, and dissection was carried down to the level of the mass, which is shown in Figure 1. Intraoperatively, the lesion measured approximately 6 × 7 cm and contained purulent material. The purulent contents were collected for culture, and the entire mass and surrounding cavity were completely excised and submitted for pathologic evaluation. Hemostasis was meticulously achieved. The wound was partially closed using nylon sutures, with placement of iodoform packing between the sutures - also demonstrated in Figure 1. The patient tolerated the procedure well.

**Figure 2:**
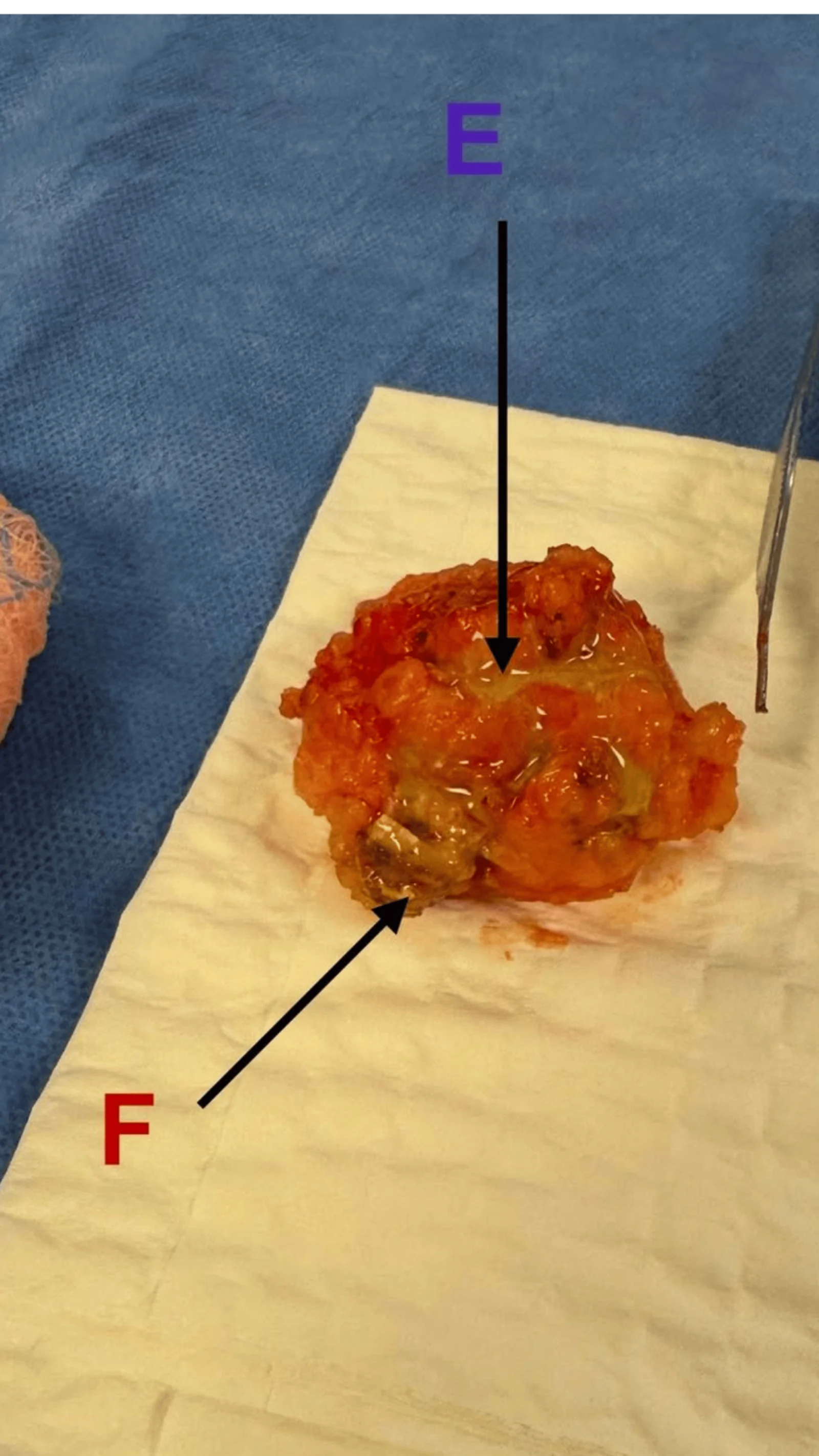
Arrows E and F demonstrate the gross pathologic specimen following serial sectioning. Arrow F highlights the area of necrotic tissue, while arrow E indicates the yellow purulent drainage expressed from the specimen.

Gross pathologic examination demonstrated a yellow-tan soft tissue specimen measuring 5.0 × 3.5 × 3.0 cm, seen in Figure 2. The specimen was inked black and serially sectioned, with yellow fluid expressed upon sectioning. Representative sections were submitted in cassettes A1-A4.

**Figure 3:**
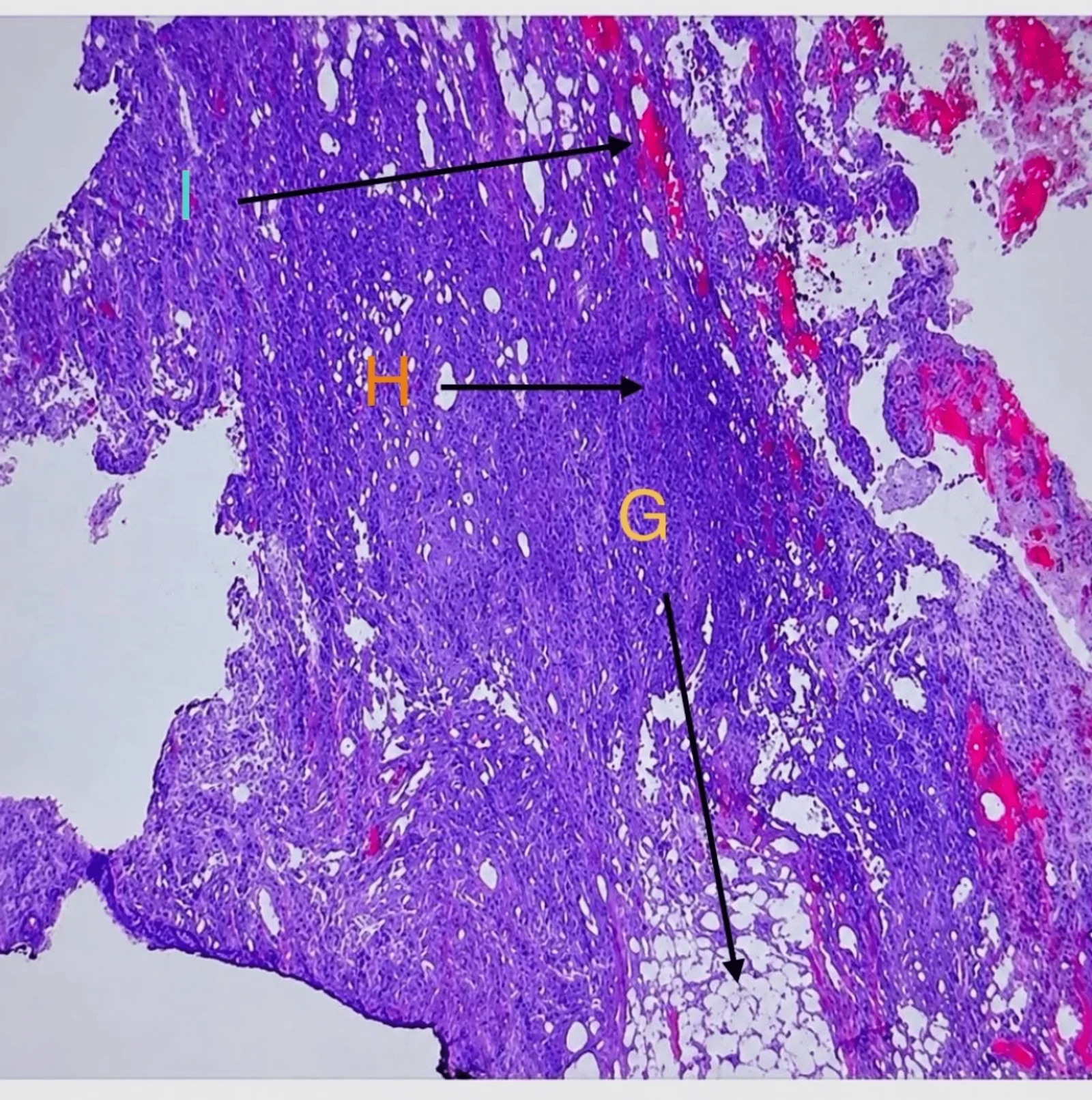
Demonstrates the hematoxylin and eosin stain at 40x magnification of the excised mass. Arrow I showcases the fat necrosis in the intensely pink stained area. Arrow H points to a darkened purple area, which demonstrates the calcification. Arrow G is exhibiting the fibroadipose tissue.

Histopathologic evaluation of the excised left buttock mass demonstrated fibroadipose tissue with focal abscess formation, acute and chronic inflammation, fat necrosis, calcification, and reactive changes as shown in Figure 3. Immunohistochemical staining with pancytokeratin (AE1/AE3) supported the diagnosis. Special stains, including acid-fast bacilli (AFB) and Gomori methenamine silver (GMS), were negative for mycobacterial and fungal organisms, respectively. No evidence of malignancy was identified.

Microbiologic evaluation of the purulent material revealed many white blood cells on Gram stain without identifiable organisms. Aerobic and anaerobic cultures demonstrated no growth after 56-72 hours. A urine pregnancy test obtained preoperatively was negative.

## Discussion

Brazilian butt lift (BBL) procedures have increased substantially in popularity over the past decade and remain among the fastest-growing cosmetic surgeries worldwide. While generally effective for gluteal contour enhancement, BBLs are associated with a wide range of complications, from minor aesthetic concerns to severe morbidity and mortality. Reported complications include seroma formation, infection, fat necrosis, contour irregularities, oil cysts, calcifications, acute and chronic inflammatory reactions, and pulmonary fat embolism. Although most complications occur during the immediate postoperative period, delayed presentations months to years after surgery are increasingly recognized.

This case describes a delayed sterile inflammatory mass that developed approximately seven years after the patient’s initial gluteal augmentation and several years after revision surgery. Histopathologic examination demonstrated fibroadipose tissue with focal abscess formation, acute and chronic inflammation, fat necrosis, calcification, and reactive changes without evidence of malignancy or active infection. Microbiologic studies, including Gram stain, aerobic and anaerobic cultures, fungal stains, and acid-fast bacilli testing, were negative. Together, these findings support a diagnosis of chronic sterile inflammatory reaction related to prior gluteal fat grafting.

Fat necrosis is a well-recognized complication of autologous fat transfer. It is primarily caused by inadequate neovascularization of transplanted adipose tissue, resulting in ischemia, adipocyte death, fibrosis, calcification, and chronic inflammation that may present as a palpable mass years after the procedure [4]. When grafted fat fails to establish an adequate blood supply, adipocyte death and liquefaction can occur, triggering a persistent inflammatory response. In some cases, sterile collections of necrotic adipose tissue may produce purulent-appearing material despite the absence of microbial growth, as observed in this patient.

A notable feature of this case is the delayed onset of symptoms. Most postoperative inflammatory complications are identified within weeks to months of surgery; however, emerging reports suggest that chronic inflammatory reactions may develop years after cosmetic augmentation procedures. Repeated fat grafting or revision surgeries may further increase this risk through cumulative tissue trauma, altered vascular architecture, and larger volumes of transplanted fat. The patient’s history of both an initial BBL and subsequent revision surgery may have contributed to chronic tissue remodeling and delayed inflammatory changes.

The differential diagnosis of a painful gluteal mass in a patient with prior cosmetic augmentation is broad and includes infectious abscess, foreign-body granuloma, fat necrosis, chronic seroma, soft tissue sarcoma, metastatic disease, and injection-related complications. Systematic reviews of autologous fat grafting have identified infection, fat necrosis, calcification, oil cyst formation, induration, and abscess formation among the most common complications, highlighting the need for careful evaluation of delayed soft tissue masses [5].

Because imaging findings are often nonspecific, definitive diagnosis frequently requires tissue sampling or surgical excision. In this case, excision served both diagnostic and therapeutic purposes by relieving symptoms and permitting comprehensive histopathologic and microbiologic evaluation. The absence of malignancy and negative infectious studies were critical in confirming the benign nature of the lesion.

This case highlights the importance of maintaining a high index of suspicion for delayed sterile inflammatory complications in patients with a history of cosmetic gluteal augmentation. Although uncommon, such reactions have been reported several years after autologous fat grafting, particularly following repeat procedures, emphasizing the need for long-term surveillance in cosmetic surgery patients [6].

As the number of BBL procedures continues to rise globally, clinicians across multiple specialties - including primary care, surgery, pathology, radiology, and emergency medicine - are increasingly likely to encounter similar presentations. Thorough evaluation is necessary to exclude infection and malignancy while avoiding unnecessary prolonged antimicrobial therapy. When clinical and radiographic findings are inconclusive, histopathologic assessment remains the diagnostic gold standard.

This report also underscores the importance of long-term postoperative surveillance and patient education. Patients should be informed that symptoms such as persistent pain, swelling, palpable masses, or contour changes may develop years after surgery and warrant medical evaluation. Early recognition may allow intervention before progression to larger symptomatic lesions requiring surgical excision.

Overall, this case broadens the spectrum of recognized long-term complications associated with autologous gluteal fat grafting and reinforces that delayed symptomatic masses warrant comprehensive evaluation, including histopathologic examination when the diagnosis remains uncertain. The combination of fat necrosis, chronic inflammation, calcification, and sterile abscess formation can closely mimic infection or malignancy, posing a significant diagnostic challenge [5]. Although these complications are uncommon, they present a clinically significant sequelae essential for accurate diagnosis. As cosmetic gluteal augmentation continues to increase in popularity, heightened awareness of these delayed complications may facilitate earlier recognition, appropriate surgical management, and avoidance of unnecessary antimicrobial therapy or extensive oncologic workup, leading to optimization of long-term patient outcomes.

## Conclusions

In the final analysis, the patient’s presentation was consistent with a chronic inflammatory subcutaneous gluteal mass with sterile abscess formation and fat necrosis occurring in the setting of prior cosmetic gluteal augmentation procedures. These chronic, sterile, inflammatory subcutaneous changes outline a section in cosmetic procedures suggesting further care and post-operative surveillance and maintenance for the patients to avoid negative outcomes and minimize patient discomfort from these procedures. This case also shows the importance of ruling out structural, infectious and malignant causes of inflammatory postoperative changes to reduce patient morbidity and negative outcomes.
